# Hardware Implementation of Lorenz Circuit Systems for Secure Chaotic Communication Applications

**DOI:** 10.3390/s130202494

**Published:** 2013-02-18

**Authors:** Hsin-Chieh Chen, Ben-Yi Liau, Yi-You Hou

**Affiliations:** 1 Department of Biomedical Engineering, Hungkuang University, Taichung 43302, Taiwan; E-Mails: hcchen@sunrise.hk.edu.tw (H.-C.C.); byliau@sunrise.hk.edu.tw (B.-Y.L.); 2 Department of Electrical Engineering, Far East University, Tainan 74448, Taiwan

**Keywords:** sliding mode controller (SMC), secure communication system, proportional-integral (PI)

## Abstract

This paper presents the synchronization between the master and slave Lorenz chaotic systems by slide mode controller (SMC)-based technique. A proportional-integral (PI) switching surface is proposed to simplify the task of assigning the performance of the closed-loop error system in sliding mode. Then, extending the concept of equivalent control and using some basic electronic components, a secure communication system is constructed. Experimental results show the feasibility of synchronizing two Lorenz circuits via the proposed SMC.

## 1. Introduction

Chaos theory is a branch of nonlinear system theory and has been intensively studied in the past four decades. In 1963, E. N. Lorenz presented the first well-known chaotic system, which was a third-order autonomous system with only two multiplication-type quadratic terms but which displayed very complex dynamic behaviors [1]. The control problems of chaos phonemes have been extensively studied over the past two decades based on its particular properties, such as broadband noise-like waveforms, difficult predictability, and sensitivity to initial condition variations, *etc.* Until now, many control methods under the assumptions of knowing the structure of nonlinearity or matching condition, have been extensively considered for the subject of chaos synchronization [2–8].

Recently, the chaos synchronization between master (transmitter) and slave (receiver) chaotic systems has been an attractive topic for its potential applications in secure communication [9–13]. Several control schemes have been developed for the synchronization of chaotic systems. Sliding mode control is a characteristic kind of variable structure system which has been a useful and distinctive robust control strategy for many kinds of engineering systems in these past two decades. By designing a switching surface and using a discontinuous control law, the trajectories of dynamic systems can be forced to slide along the fixed sliding manifold. The sliding mode control technique has been successfully applied to synchronization of chaotic system [5,6,11]. Work [14] proposed a proportional-integral (PI) control scheme based on SMC technique-based to solve the synchronization problem of unified chaotic systems. The proposed PI controller is used to guarantee the synchronization between the transmitter and the receiver in secure communication systems.

To verify the above systems performance, in this paper a SMC-based chaotic secure communication system, which includes two chaotic Lorenz circuits (transmitter and receiver) and a sliding mode controller, is realized by using some electronic components containing operational amplifiers (OPAs), resistors and capacitors.

## 2. Problem Formulation and Main Results

The aim of this paper was to utilize the unpredictable characteristics of chaos signals, such as broadband noise-like waveform, prediction difficulty and sensitivity to initial condition variations, to construct a secure communication system. Now we consider the following Lorenz circuits, which are typical chaotic systems that have been thoroughly studied [14].

Master Lorenz chaotic circuit:

(1) $$\begin{array}{ll} {\dot{x}}_{m1}(t) & =(25\alpha +10)(x_{m2}(t)-x_{m1}(t))\\ {\dot{x}}_{m2}(t) & =(28-35\alpha )(x_{m1}(t)+(29\alpha -1)x_{m2}(t)-x_{m1}(t)x_{m3}(t)+p(t)\\ {\dot{x}}_{m3}(t) & =x_{m1}(t)x_{m2}(t)-\frac{\left(8+\alpha \right)}{3}x_{m3}(t) \end{array}$$

Slave Lorenz chaotic circuit:

(2) $$\begin{array}{ll} {\dot{x}}_{s1}(t) & =(25\alpha +10)(x_{s2}(t)-x_{s1}(t))\\ {\dot{x}}_{s2}(t) & =(28-35\alpha )x_{s1}(t)+(29\alpha -1)x_{s2}(t)-x_{m1}(t)x_{s3}(t)+u(t)\\ {\dot{x}}_{s3}(t) & x_{m1}(t)x_{s2}(t)-\frac{\left(8+\alpha \right)}{3}x_{s3}(t) \end{array}$$

Obviously, Equations (1) and (2) becomes the original Lorenz system for *α* = 0, where *ẋ_m_* and *ẋ_s_* denote the derivative of *x_m_* and *x_s_* with respect to time *t*, *u*(*t*) is the controller output used to synchronize the master and slave systems Equations (1) and (2), and *p*(*t*) is the embedded message bounded by:

(3) $$\begin{array}{cc} \left|p(t)\right|\leq \psi , & \psi \end{array}>0$$

The control goal is for the two Lorenz chaotic systems Equations (1) and (2) to be synchronized such that the resulting error vector satisfies:

(4) $$\begin{array}{cc} \underset{t\to \infty }{\lim}\left\|e(t)\right\|=\underset{t\to \infty }{\lim}\left\|x_{mi}(t)-x_{si}(t)\right\|=0, & i=1,2,3 \end{array}$$

The error vectors and error dynamics are defined as:

(5) $$\begin{array}{ll} e_{1}(t) & =x_{m1}(t)-x_{s1}(t)\\ e_{2}(t) & =x_{m2}(t)-x_{s2}(t)\\ e_{3}(t) & =x_{m3}(t)-x_{s3}(t) \end{array}$$

and:

(6) $$\begin{array}{ll} {\dot{e}}_{1}(t) & ={\dot{x}}_{m1}(t)-{\dot{x}}_{s1}(t)\\ {\dot{e}}_{2}(t) & ={\dot{x}}_{m2}(t)-{\dot{x}}_{s2}(t)\\ {\dot{e}}_{3}(t) & ={\dot{x}}_{m3}(t)-{\dot{x}}_{s3}(t) \end{array}$$

Then, the following error dynamics are obtained:

(7) $$\begin{array}{ll} {\dot{e}}_{1}(t) & =(25\alpha +10)(e_{2}(t)-e_{1}(t))\\ {\dot{e}}_{2}(t) & =(28-35\alpha )e_{1}(t)+(29\alpha -1)e_{2}(t)-x_{m1}e_{3}(t)+p(t)-u(t)\\ {\dot{e}}_{3}(t) & =x_{m1}e_{2}(t)-\frac{\left(8+\alpha \right)}{3}e_{3}(t) \end{array}$$

To stabilize the error dynamics Equations (7) and achieve synchronization, two basic steps are used: first, an appropriate switching surface is selected such that the sliding motion on the sliding manifold is stable and ensures lim*_t_*_→∞_ ‖*e*(*t*)‖ = 0; second, a SMC law which guarantees the existence of the sliding mode *s*(t) = 0 is established. To guarantee the asymptotic stability of the sliding mode, the PI switching surface *s*(t) is defined as:

(8) $$s(t)=e_{2}(t)+\int_{0}^{t}((25\alpha +10)e_{1}(\tau )+x_{m1}e_{3}(\tau )+\beta e_{2}(\tau ))d\tau$$

where *β* > 0 is given.

Having established the appropriate switching surface Equations (8), as described above, the next step is to design a SMC scheme to drive the system trajectories onto the sliding mode *s*(*t*) = 0. This study proposes the following SMC:

(9) $$\begin{array}{cc} u(t)=u_{1}(t)+\eta \psi [\text{sign}(s(t))], & \eta >1 \end{array}$$

where *u*_1_(*t*) = (38 + 10*α*)*e*_1_(*t*) + (29*α* − 1 + *β*)*e*_2_(*t*).

After design the control to ensure lim*_t_*_→∞_ ‖*E*(*t*)‖ = lim*_t_*_→∞_ ‖[_1_(*t*) *e*_2_(*t*) *e*_3_ (*t*)]‖ = 0. We have the following fact:

(10) $$\begin{array}{lll} {\dot{e}}_{1}(t)=(25\alpha +10)(e_{2}(t)-e_{1}(t))\to 0 & & {\dot{e}}_{1}(t)\to 0\\ {\dot{e}}_{2}(t)=(28-35\alpha )e_{1}(t)+(29\alpha -1)e_{2}(t)-x_{m1}e_{3}(t)+p(t)-u(t)\to 0 & \Rightarrow & {\dot{e}}_{2}(t)=0+p(t)-u(t)\to 0\\ {\dot{e}}_{3}(t)=x_{m1}e_{2}(t)-\frac{\left(8+\alpha \right)}{3}e_{3}(t)\to 0 & & {\dot{e}}_{3}(t)\to 0 \end{array}$$

Then, we can infer that:

(11) $$\underset{t\to \infty }{\lim}(p(t)-u(t))=0$$

which means that the message *p*(*t*) can be approximated by the control *u*(*t*). From the works [15,16], the control input *u*(*t*) can be approximated by the following continuous equivalent control *u_eq_*(*t*):

(12) $$u_{eq}(t)=u_{1}(t)+\eta \psi \left[\frac{s(t)}{|s(t)|+\sigma }\right]$$

where *σ* is an arbitrarily small positive constant. When we choose a small enough *σ*, then Equation (12) will arbitrarily approach to Equations (9) and input message *p*(*t*) can be recovered by Equation (12).

## 3. Experimental Results

**Figure 1.**
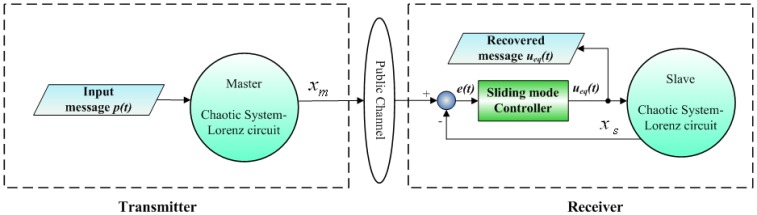
Block diagram of SMC-based scheme secure communication system.

The preceding SMC scheme of synchronization is applied to establish chaotic secure communication systems. Figure 1 illustrates the proposed communication system that consists of a transmitter and a receiver [aster and slave Lorenz circuits (for *α* = 0), respectively]. The input message *p*(*t*) is embedded into the chaotic transmitter and the state of the master Lorenz system is simultaneously transmitted to the receiver. The equivalent SMC synchronization scheme of Equation (12) is given in the receiver. From the discussion in the section above, it ensures that the input message *p*(*t*) can be completely recovered on the receiver side using the equivalent controller Equation (12), if the synchronization between the transmitter and the receiver can be achieved.

**Figure 2.**
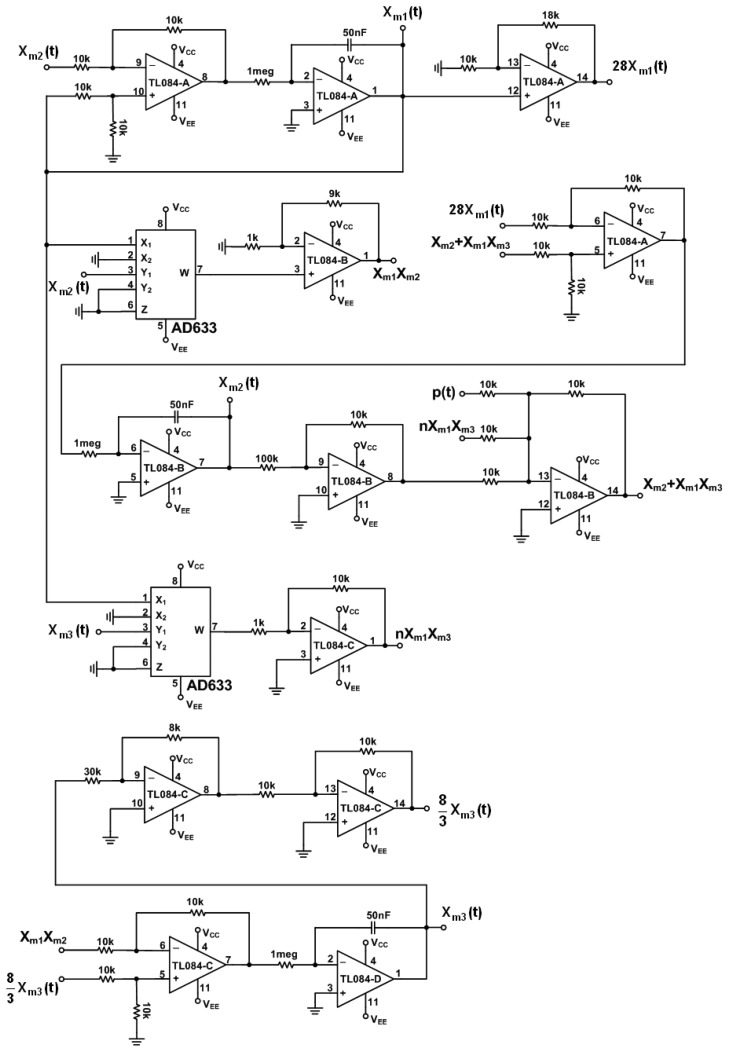
Electronic implementation of the master Lorenz circuit.

**Figure 3.**
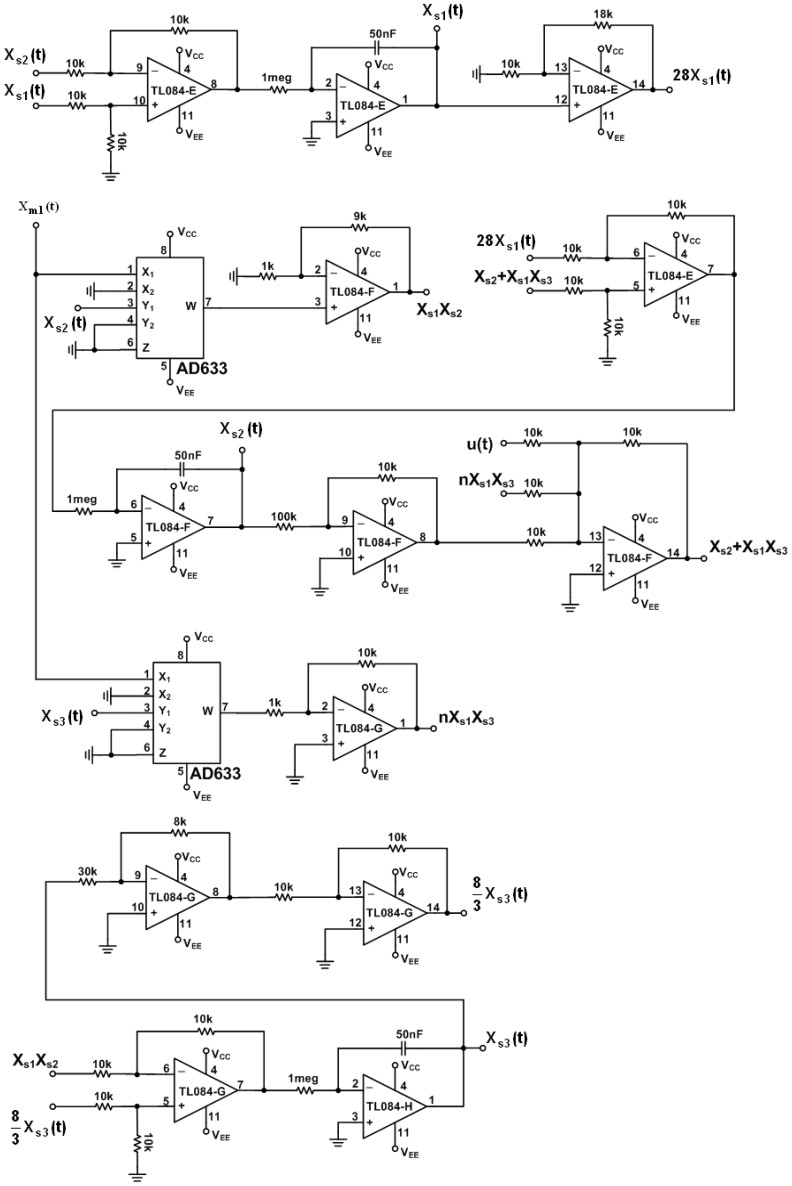
Electronic implementation of the slave Lorenz circuit.

In the following, we use simple electronic components: OPAs, resistors and capacitors to implement the presented secure communication system. In order to speed up the dynamic response of chaotic Lorenz circuit, we rescale the systems Equations (1) and (2) by a new time scale $\tau =\frac{t}{k}$, and then we have the following systems, respectively:

(13) $$\begin{array}{ll} {\dot{x}}_{m1}(\tau ) & =k[10(x_{m2}(\tau )-x_{m1}(\tau ))]\\ {\dot{x}}_{m2}(\tau ) & =k[28x_{m1}(\tau )-x_{m2}(\tau )-k_{1}x_{m1}(\tau )x_{m3}(\tau )+p(\tau )]\\ {\dot{x}}_{m3}(\tau ) & =k[k_{1}x_{m1}(\tau )x_{m2}(\tau )-\frac{8}{3}x_{m3}(\tau )] \end{array}$$

(14) $$\begin{array}{ll} {\dot{x}}_{s1}(\tau ) & =k[10(x_{s2}(\tau )-x_{s1}(\tau ))]\\ {\dot{x}}_{s2}(\tau ) & =k[28x_{s1}(\tau )-x_{s2}(\tau )-k_{1}x_{m1}(\tau )x_{s3}(\tau )+u(\tau )]\\ {\dot{x}}_{s3}(\tau ) & =k[k_{1}x_{m1}(\tau )x_{s2}(\tau )-\frac{8}{3}x_{s3}(\tau )] \end{array}$$

where *ẋ_m_* and *ẋ_s_* denote the derivative of *x_m_* and *x_s_* with respect to time τ, respectively. *k* and *k*_1_ is a scaling factor. Practical circuits of the chaotic master and slave Lorenz systems with *k* = 20 and *k*_1_ = 10 and supplied voltages ±15 V are shown in Figures 2 and 3, respectively.

**Figure 4.**
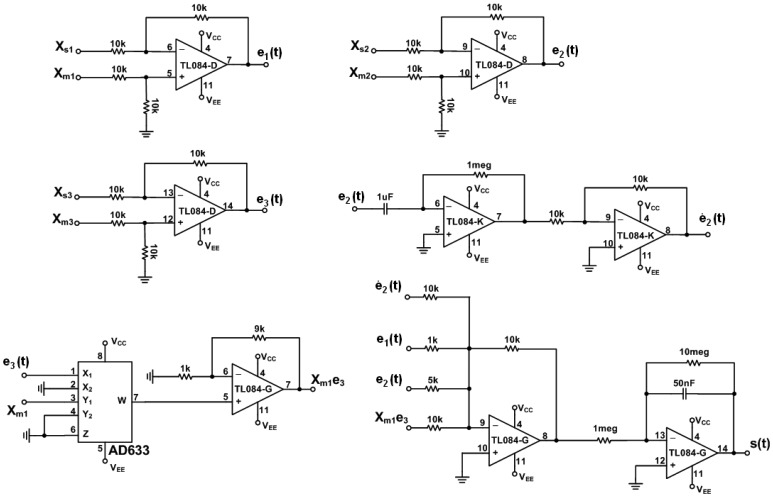
Electronic implementation of the error dynamics *e*(t) and the switch surface *s*(t) circuit.

**Figure 5.**
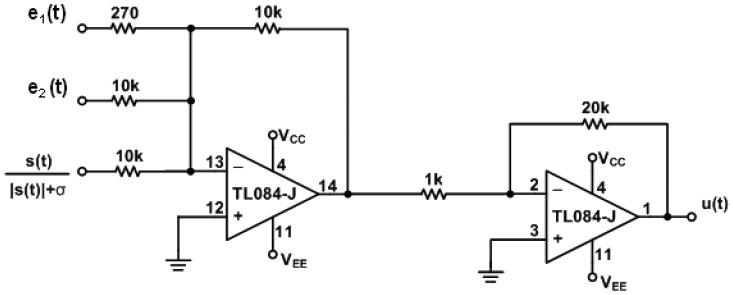
Electronic implementation of *u_eq_*(*t*) circuit.

**Figure 6.**
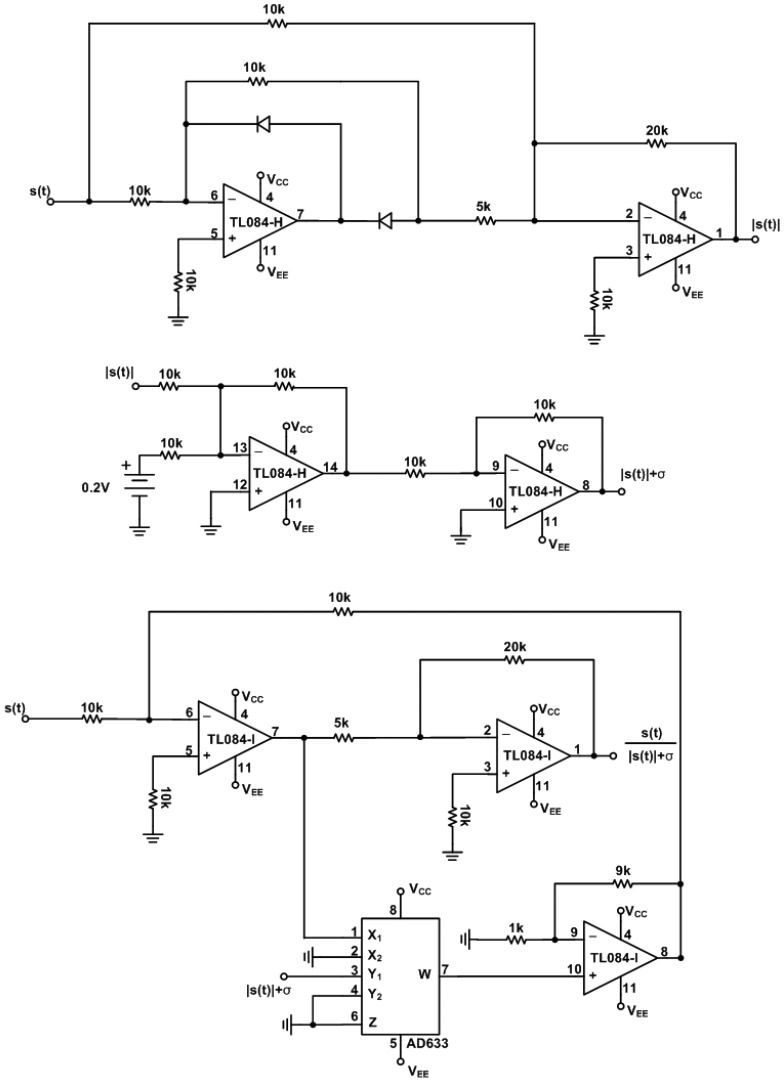
Electronic implementation of $\eta \psi \left[\frac{s(t)}{|s(t)|+\sigma }\right]$circuit.

The circuit of error dynamics *e*(*t*) and the switch surface *s*(*t*) circuit are shown in Figure 4. The circuits of the continuous equivalent control *u_eq_*(*t*) Equations (12) is shown in Figures 5 and 6. In order to demonstrate the chaotic secure communication, the embedded message *p*(*t*) is specified as a sine wave (1 V, 5 Hz) in the transmitter.

**Figure 7.**
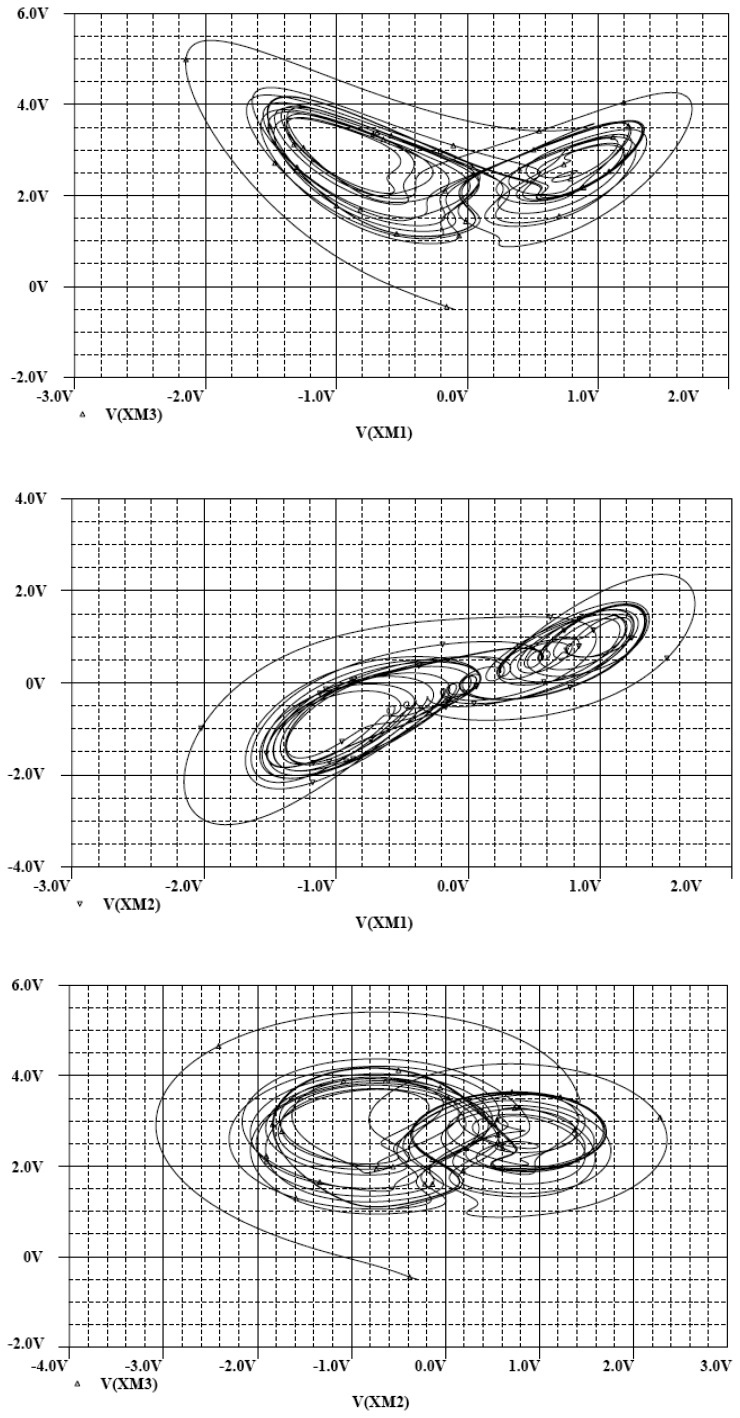
The trajectories of the Lorenz system.

**Figure 8.**
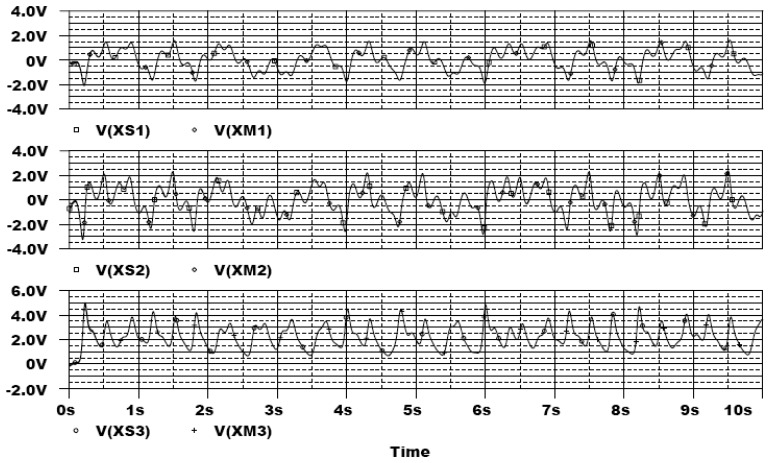
Experimental results of synchronization between state *x_m_* and state *x_s_*.

**Figure 9.**
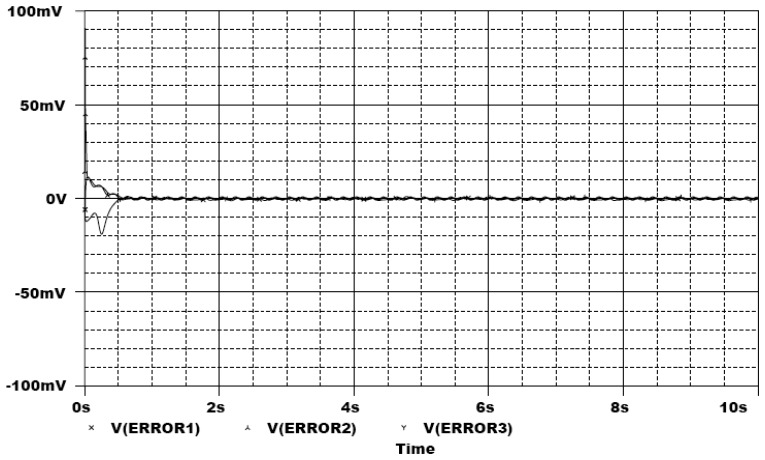
Experimental results of errors between state *x_m_* and state *x_s_*.

**Figure 10.**
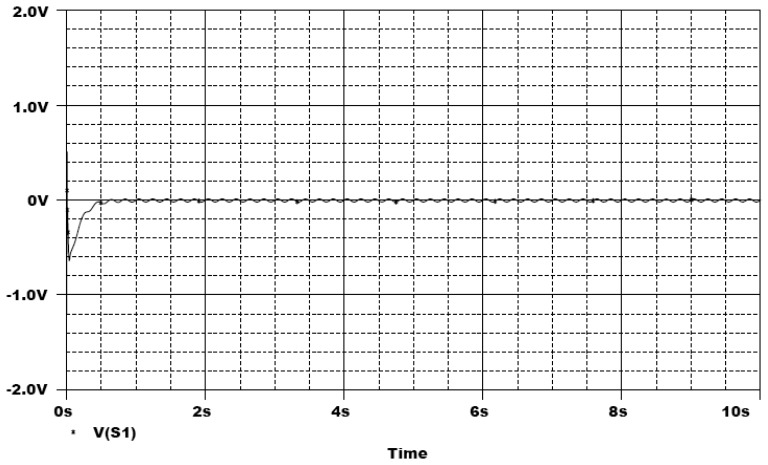
Experimental result of switch surface *s*(t).

**Figure 11.**
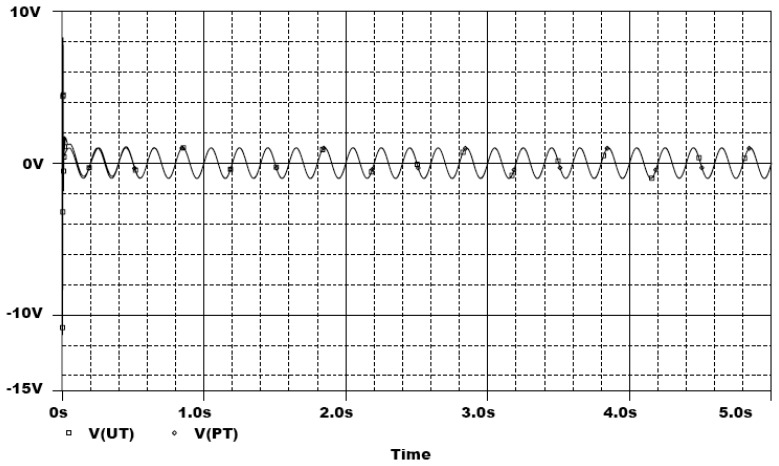
Experimental results of control input *u_eq_*(*t*) and input message *p*(t).

In the following, the commercial electronic circuit simulation software Orcad/PSpice 9.0 is used. Figure 7 shows the trajectories of the Lorenz system. Figure 8 shows the experimental results of synchronization between state *x_m_* and state *x_s_*. Figure 9 shows the experimental results of errors between state *x*_m_ and state *x*_s_. Figure 10 shows the experimental result of switch surface *s*(t). From these figures, we can observe that the switching surface *s*(t) approaches zero within 0.5 s and the synchronization errors approach zero after 0.5 s, and then both the master and slave are synchronous. Figure 11 shows the experimental results of the continuous equivalent control *u_eq_*(*t*) and input message *p*(t). From this figure, we can observe that the input message *p*(t) (sine wave: 1 V, 5 Hz) can be successful recovered.

## 4. Conclusions

This study has been proposed to ensure the synchronization between the master and the controlled slave Lorenz chaotic systems via a sliding mode controller. Furthermore, the proposed scheme has been also successfully applied to a secure communication system. Some basic electronic circuits are used to implement the SMC-based secure communication system. The experimental results verify that the methods are correct and practical.
